# Supplementary material for: The Gambling Habits of University Students in Aragon, Spain: A Cross-Sectional Study
Source: Int J Environ Res Public Health. 2022 Apr 9;19(8):4553. doi: 10.3390/ijerph19084553 (PMC9024798; doi:10.3390/ijerph19084553)
Supplement: Supplementary file 1 [file ijerph-19-04553-s001.zip › ijerph-1650713-supplementary.pdf]

Survey on gambling and betting behaviours

A research team from the University of Zaragoza is going to carry out a study in relation to gambling and betting behaviours in university students. Your participation will be anonymous, and the data collected in the study will be used for research purposes only.

- Would you like to participate in the study? (YES / NO)

First, we would like to know a little more about you:

1. How old are you? _____

2. Which is your gender? (MALE / FEMALE / OTHER)

3. Where do you live? (ZARAGOZA-CAPITAL / ZARAGOZA-PROVINCE / HUESCA-CAPITAL / HUESCA-PROVINCE / TERUEL-CAPITAL / TERUEL-PROVINCE)

4. How much money do you have for your expenditures? (€0-€100 / €101-€400 / €401-€600 / €601-€1,000 / more than €1,001)

5. Are you interested in sports? (NOT AT ALL / A LITTLE / QUITE INTERESTED / A LOT)

6. Do you do sport every week? (YES / NO)

7. What degree are you studying? _____

8. Which year are you studying? (1st / 2nd / 3rd / 4th / 5th / 6th)

9. Which area of knowledge does your degree belong to? (ARTS AND HUMANITIES / SCIENCES / HEALTH SCIENCES / SOCIAL AND JURIDIC SCIENCES / ENGINEIRING AND ARCHITECTURE / SPORTS SCIENCES)

10. Which is your district? (CENTRO / CASCO HISTÓRICO / LAS FUENTES / SAN JOSÉ / TORRERO / UNIVERSIDAD / CASABLANCA / DELICIAS / OLIVER-VALDEFIERRO / MIRALBUENO / LA ALMOZARA / ACTUR / EL RABAL / SANTA ISABEL)

Micro-transactions

1. Have you ever paid money for a micro-transaction in a smartphone, tablet or computer game? *In some games, there is the option of introducing real money in exchange for different advantages, such as the "boxes".* (YES / NO)

2. If so, how much money have you spent in total? (€0-€10 / €11-20 / €21-€30 / more than €30)

Knowledge on gambling options, betting advertising and prevention messages

1. Could you locate some betting shops? (NO / YES, A FEW / YES, SOME OF THEM / YES, MANY OF THEM)

2. Do you know the name of any betting website or app? (NO / YES, A FEW / YES, SOME OF THEM / YES, MANY OF THEM)

3. How often do you receive publicity about bets? *It can appear on billboards, surfing the Internet, on television, radio…* (NEVER / SELDOM / SOMETIMES / OFTEN / VERY OFTEN / DAILY)

4. Have you ever received any information preventing you from betting? (YES / NO)

5. If so, who provided the information? *You can mark more than one option.* (THE INTERNET / PARENTS OR RELATIVES / SCHOOL, HIGH SCHOOL OR UNIVERSITY / OTHER SOURCES)

Sports bets

1. Have you ever gambled on sporting events? *It could be football match, a race, etc.* (YES / NO)

2. Did you receive a welcome bonus when you did the first bet? (YES / NO)

3. If so, what was the amount of the bonus? ____

4. How important was for you this bonus for betting for the first time? [0 = not important at all – 10 = very important]

5. Have you ever bet on a sporting event which was happening in that moment? (YES / NO)

6. Which sporting events have you bet on? *Mark as many options as you need.* (FOOTBALL / BASKETBALL / HANDBALL / TENNIS / HORSE RACES / DOG RACES / CYCLING / MOTORING / MOTORCYCLING)

7. Currently, how often do you bet on live sporting events? (ON SPECIFIC OCCASIONS / ONCE PER MONTH / ONCE EVERY TWO WEEKS / ONCE PER WEEK / MANY TIMES PER WEEK / EVERY DAY)

8. Have you ever used the promotions or advantages that the app offers? *Double bets, special bets, promotions…* (YES, VERY OFTEN / YES, SOMETIMES / NO)

9. How much do you spend on average every week on sports bets? ____

10. How much do you think you have spent in total on sports bets? _____

Betting shops

By betting shops we refer to those establishments that can be found on the street and that allow you to play bingo, roulette, card games and/or place bets on sporting events

(football games, basketball, races ...) in a face-to-face way. Some examples are: *El Dorado, Arabet, Sportium, Alea, Luckia...*

1. Have you ever entered to one of this establishments? (YES / NO)

2. How old were you the first time you entered to a betting shop? ____

3. Which was your main reason for entering a betting shop for the first time? (TO ACCOMPANY A FRIEND / TO TRY MY LUCK / TO EARN SOME MONEY / CURIOSITY)

4. Currently, how often do you go to betting shops? (ALMOST NEVER / ON SPECIFIC OCCASIONS / ONCE PER MONTH / ONCE EVERY TWO WEEKS / ONCE PER WEEK / MANY TIMES PER WEEK / EVERY DAY)

5. How often do they ask you to show your identification in the betting shop? (ALWAYS / NORMALLY / OFTEN / SOMETIMES / OCCASIONALLY / RARELY / NEVER)

6. Have you ever gambled on a betting shop? (YES / NO)

7. What games have you played? *Mark as many options as you need.* (SPORTS BETS / ROULETTE / INSTANT LOTTERY / POKER / BLACKJACK / OTHER CARDS GAMES / BINGO / SLOT MACHINE / OTHER-SPECIFY)

8. How much money do you spend, on average, on betting shops every week? ____

9. Have you ever earned a substantial amount of money on a betting shop? (YES / NO)

10. Mark the main emotion you feel in every moment:

	Sad	Enthusiastic	Nervous	Disappointed	Satisfied	Indifferent	Bored	None

Before gambling								
While gambling								
After gambling								

11. When I gamble, I normally do it… *Mark as many options as you need.* (IN THE MORNING / IN THE AFTERNOON / AT NIGHT)

12. How often do you think about gambling on betting shops? (NEVER / RARELY / ON SPECIFIC OCCASIONS SUCH AS WEEKENDS / OFTEN / DAILY)

Betting websites

By betting websites, we refer both to the Internet pages themselves and the applications that can be had on smartphones, tablets, and computers through which real money is wagered. Examples of the main pages are: *William Hill, Bet365, Codere, 888, Wanabet, Sportium online...*

1. Have you ever visited a betting website? (YES / NO)

2. How old were you the first time you visited a betting website? ____

3. Do you have an account on any of these websites? (NO / YES, ONE / YES, MORE THAN ONE)

4. When you registered on the website, did you use your real identification (DNI, name, etc.)? (YES / NO / I DID NOT HAVE TO INTRODUCE ANY PERSONAL DATA)

5. Have you ever gambled on the Internet? (YES / NO)

6. Did you receive a welcome bonus when you did the first bet? (YES / NO)

7. If so, what was the amount of the bonus? ____

8. How important was for you this bonus for betting for the first time? [0 = not important at all – 10 = very important]

9. Currently, how often do you visit betting websites? (ALMOST NEVER / ON SPECIFIC OCCASIONS / ONCE PER MONTH / ONCE EVERY TWO WEEKS / ONCE PER WEEK / MANY TIMES PER WEEK / EVERY DAY)

10. What games have you played? *Mark as many options as you need.* (SPORTS BETS / ROULETTE / INSTANT LOTTERY / POKER / BLACKJACK / OTHER CARDS GAMES / BINGO / SLOT MACHINE / OTHER-SPECIFY)

11. How much money do you spend, on average, on betting websites every week? ____

12. Have you ever earned a substantial amount of money on a betting website? (YES / NO)

13. Mark the main emotion you feel in every moment:

	Sad	Enthusiastic	Nervous	Disappointed	Satisfied	Indifferent	Bored	None
Before gambling								
While gambling								

After gambling								

14. When I gamble on the Internet, I normally do it… *Mark as many options as you need.* (IN THE MORNING / IN THE AFTERNOON / AT NIGHT)

12. How often do you think about gambling on betting websites? (NEVER / RARELY / ON SPECIFIC OCCASIONS SUCH AS WEEKENDS / OFTEN / DAILY)

Opinions on gambling and its regulation

1. Do you have any friend who gambles regularly? (YES / NO)

2. Do you have any acquaintance who has earned money gambling? (YES, A SUBSTANTIAL AMOUNT OF MONEY / YES, SMALL AMOUNTS OF MONEY / NO)

3. Do you know about the existence of a register where people can self-ban themselves for entering to betting shops and websites? (YES / NO)

4. Do you agree with the following statements? (YES / NO)

- Young people are frequently betting on sports
- Young people are frequently gambling on betting shops
- Young people are frequently gambling on the Internet
- There are few betting shops
- Bets are a normal activity

5. What do you think about the regulation of betting advertisements? (IT IS WELL REGULATED / IT SHOULD BE MORE RESTRICTIVE / BETTING ADVERTISEMENTS SHOULD BE BANNED)

6. Have you ever bet money? *This includes betting shops, websites, and sports bets*. (YES / NO)

7. How dangerous do you consider gambling, both face-to-face and online? [0 = not dangerous at all, 10 = very dangerous]

8. Which is your main reason to gamble? *I mainly gamble…* (TO SPEND TIME WITH MY FRIENDS AND FAMILY / TO EARN MONEY / BECAUSE IT MAKES ME FEEL GOOD / TO ENTERTAIN MYSELF / BECAUSE THAT'S WHAT MY FRIENDS DO / BECAUSE IT HELPS ME WHEN I'M FEELING DOWN OR NERVOUS / OTHER-SPECIFY)

Brief Questionnaire of Pathological Gambling + Risk factors ad hoc scale

1. Do you believe that you have or have had problems with gambling? (YES / NO)

2. Have you ever felt guilty for gambling or for what happens while you gamble? (YES / NO)

3. Have you ever tried to stop gambling and you have not been able to do so? (YES / NO)

4. Have you ever taken money from home to gamble or to pay your gambling debts? (YES / NO)

5. Have you gambled more than you intended to? (YES / NO)

6. Have you ever neglected your obligations (e.g., family, work or school) because of gambling? (YES / NO)

7. While gambling, have you ever lost track of time? (YES / NO)

8. Have you been told (or have you thought) of asking for professional help? (YES / NO)
